# High-Temperature Oxidation Behavior of TiN-, Cr-, and TiN–Cr PVD-Coated Zircaloy 4 Alloy at 1200 °C

**DOI:** 10.3390/ma18081692

**Published:** 2025-04-08

**Authors:** Yan-Yu Tang, Yin-Lin Chang, Wen Luo, De-Wen Tang

**Affiliations:** 1 School of Nuclear Science and Technology, University of South China, Hengyang 421001, China; tycan24@163.com (Y.-Y.T.); 2010000875@usc.edu.cn (W.L.); 2 Hunan Provincial Key Laboratory of Emergency Safety Technology and Equipment for Nuclear Facilities, University of South China, Hengyang 421001, China; 2010001480@usc.edu.cn

**Keywords:** zirconium alloy, high-temperature oxidation behavior, microstructure, TiN coating, Cr coating, TiN–Cr coating

## Abstract

Zirconium alloys are essential materials for nuclear fuel cladding. During a loss-of-coolant accident (LOCA), zirconium alloy cladding can oxidize in high-temperature steam (>1000 °C), generating hydrogen and releasing significant heat. Without timely emergency actions, this can result in hydrogen explosions or nuclear leakage. In this study, titanium nitride (TiN), chromium (Cr), and TiN–Cr composite coatings were deposited on the surface of Zr-4 alloy using the magnetron sputtering method. The coatings’ surface and cross-sectional morphologies were examined using scanning electron microscopy (SEM), and their phase structures were analyzed with X-ray diffraction (XRD). The mechanical properties were evaluated using scratch tests, and their resistance to high-temperature steam oxidation was tested in a tube furnace connected to a steam generator. The results showed that the TiN, Cr, and TiN–Cr coatings exhibited strong adhesion to the Zr-4 substrates, with distinct interfaces and pure phase structures. After high-temperature steam oxidation, cracks appeared on the surfaces of the TiN, Cr, and TiN–Cr coatings, likely due to differences in the thermal expansion coefficients of TiO_2_, Cr_2_O_3_, and residual Cr layers. These cracks created pathways for the oxidizing medium, potentially leading to the oxidation of the substrate or inner layers of the composite coatings. For the Cr and TiN–Cr coatings, despite cracking of the Cr layer and melting of the TiN layer at high temperatures, the residual Cr layer effectively restricted oxygen diffusion into the Zr-4 substrate. This study suggests that layers with low melting points, such as TiN, are unsuitable for composite coatings in high-temperature applications. However, adding a Cr layer on top of the TiN layer to form a TiN–Cr composite coating improves adhesion between the coating and the substrate. The TiN–Cr composite coating functions as an effective diffusion barrier at temperatures up to 1200 °C, comparable to a pure Cr coating.

## 1. Introduction

Zirconium alloys are widely used as materials for nuclear fuel cladding due to their excellent corrosion resistance, mechanical strength, low-neutron-absorption cross section, and overall reliable performance [1,2]. However, the Fukushima nuclear accident highlighted the insufficient high-temperature oxidation resistance of these alloys [3,4]. During a loss-of-coolant accident, zirconium alloy cladding can rapidly heat up, triggering oxidation reactions with high-temperature water vapor. This process produces significant amounts of hydrogen and heat, posing severe risks to nuclear safety. To address these challenges, the concept of accident-olerant fuel (ATF) has been proposed and has received increasing attention [1,2,3,4]. These ATF materials, which are based on zirconium alloys, aim to improve both mechanical strength and high-temperature oxidation resistance, thereby enhancing safety margins.

In recent years, various protective coatings for zirconium alloys have been developed to meet ATF criteria [5,6,7,8,9,10]. Numerous technologies and coating types have been explored, and candidate coatings are summarized in Table 1 [8,11,12,13,14,15,16,17,18,19,20,21,22,23]. Park et al. [3,4] used arc ion plating to deposit a chromium (Cr) layer on Zircaloy 4 as an oxidation-resistant layer for ATF cladding. This Cr layer remained intact in a high-temperature steam environment at 1473 K for 2000 s, demonstrating the effectiveness of arc ion plating as a corrosion protection method for ATF claddings. Michau et al. [5] investigated DLI-MOCVD (direct liquid injection of metal organic precursors) to develop coatings, including metallic Cr, chromium carbides (CrxCy), and mixed carbides (CrxSizCy). These coatings postponed catastrophic oxidation to higher temperatures and delayed complete substrate oxidation for over two hours at 1473 K. Li et al. [6,7,8] prepared MAX phase coatings such as Ti_2_AlC, Ti_3_SiC_2_, and Ti_3_AlC_2_ on 316L stainless steel using DC magnetron sputtering. They observed that oxides formed within the Ti_2_AlC and Ti_3_AlC_2_ coatings in water vapor environments at 750 °C. Additionally, the significant difference in thermal expansion between Ti_3_SiC_2_ and the substrate negatively affected coating quality control. Anasori et al. [9] studied the protective effect of Ti_2_AlC coatings with a TiC diffusion barrier layer on substrates exposed to high-temperature steam oxidation at 800 °C. They found that Ti_2_AlC–TiC composite coatings exhibited superior high-temperature oxidation resistance compared to single-layer Ti_2_AlC coatings. The TiC transition layer likely inhibited Al diffusion into the substrate during high-temperature oxidation, maintaining a high Al content in the coating. Incorporating a transition layer between the coating and substrate effectively improves the interface structure, increases interface dislocation density, and significantly enhances the bonding strength and performance of the coating.

This study was a detailed investigation into the high-temperature oxidation behavior and microstructural characteristics of zirconium alloys coated with TiN, Cr, and TiN–Cr using the physical vapor deposition (PVD) technique. The coatings were applied to Zr-4 alloy plates via direct current magnetron sputtering. After deposition, the coatings’ key attributes, including thickness, microstructure, and morphology, were thoroughly examined. The high-temperature oxidation performance of the TiN, Cr, and TiN–Cr coatings was then systematically evaluated. Oxidation-induced weight gain and the microstructural properties of the coatings were analyzed using X-ray diffraction (XRD), scanning electron microscopy (SEM), and energy-dispersive spectroscopy (EDS). The results are discussed and interpreted, culminating in a comparative analysis of high-temperature oxidation resistance among the TiN-, Cr-, and TiN–Cr-coated zirconium alloys.

## 2. Experimental Procedures

### 2.1. Sample Preparation

Zircaloy 4, a widely used cladding material in nuclear reactors, was selected as the substrate for this study. The chemical composition of the Zr-4 alloy is presented in Table 2. Substrate samples with dimensions of 20 × 20 × 2 mm were precision-cut using electric spark wire cutting technology. To facilitate the coating deposition process, a through-hole with a diameter of 2 mm was machined into the surface of each sample. To minimize the influence of heat-affected zones on the zirconium alloy, the samples were lightly trimmed with sandpaper. The substrates were then sequentially polished using diamond sandpapers with grit sizes of 100, 300, 600, 800, 1000, and 1200. Polishing continued until the surfaces attained a mirror-like finish, using a mechanical polisher and chromium oxide polishing powder with particle sizes below 0.1 μm. After polishing, the zirconium alloy specimens were thoroughly cleaned with deionized water, followed by ultrasonic cleaning in anhydrous ethanol for 15 min to eliminate any residual contaminants. Once dried, the specimens were ready for the coating deposition process.

The coatings were deposited using a magnetron sputtering system, a commonly employed PVD (physical vapor deposition) technique, which allows for precise and controlled coating deposition on the substrate. In this study, high-purity chromium (Cr) and titanium nitride (TiN) targets were used to deposit TiN, Cr, and TiN–Cr coatings onto zirconium alloy substrates. The PVD process involved placing the substrates in a vacuum chamber, which was first evacuated to a high vacuum level to reduce the presence of contaminants. Subsequently, high-purity argon gas was introduced into the chamber, acting as the protective working gas for the sputtering process. The chamber pressure was maintained at approximately 2 Pa throughout the deposition for all three coating types to ensure consistent sputtering conditions.

To facilitate uniform deposition, the substrates were positioned approximately 75 mm from the targets within the chamber. The substrates were mounted on a custom tool that enabled both rotation and orbital motion during sputtering. This setup ensured uniform coating thickness by allowing the substrates to experience the deposition flux from various angles during the process. This motion was crucial for achieving a homogeneous coating on multiple specimens simultaneously in a single sputtering cycle.

For the TiN coating, a high-purity titanium (Ti) target was used, and the deposition was carried out under nitrogen gas atmosphere at a temperature of 350 °C. The process parameters for TiN deposition included an arc current of 80 A, a substrate bias of −100 V, and a deposition time of 10 h, all controlled using a QX-400 ultrahigh-vacuum multifunctional coating machine (Chengdu Qixing Vacuum Coating Technology Co., Ltd, Chengdu, China). These conditions were optimized to ensure high-quality TiN coatings with good adhesion and desired structural properties.

Similarly, the Cr coating was deposited under the same conditions as the TiN coating, but using a high-purity Cr target instead. For the TiN–Cr composite coating, a sequential deposition process was employed, where the TiN layer was deposited first, followed by the Cr layer. Each layer was deposited for 5 h, maintaining the same temperature, bias, and vacuum conditions to ensure the formation of a well-adhered and homogeneous composite coating. The other sputtering parameters are shown in Table 3.

### 2.2. High-Temperature Oxidation Testing

High-temperature oxidation (HTO) performance is a critical attribute for ensuring the safety of nuclear fuel cladding tubes during a loss-of-coolant accident. This study evaluated the HTO performance of various coatings applied to the surface of zirconium alloy using a KBF1600 furnace (Nanjing University Instrument Factory, Nanjing, China). The heating protocol consisted of an initial rapid temperature increase from room temperature to 300 °C, followed by a gradual heating of the specimens to 1200 °C at a rate of 20 °C per minute. Prior to heating, the initial weight of each coated Zr-4 specimen, along with that of an uncoated Zr-4 reference specimen, was carefully recorded. Once the furnace temperature had reached and stabilized at 1200 °C, the specimens were introduced into the furnace. After 4 h at 1200 °C, the specimens were retrieved and allowed to air-cool to room temperature. The weight gain due to oxidation was calculated by determining the difference in specimen weight before and after the heating process. For comparison, an uncoated Zr-4 specimen underwent the same heating and weighing protocol.

### 2.3. Experimental Methods

The morphology of the fracture surface and inner surface of the oxide film formed on the deposited coatings was characterized using an EVO-18 high-resolution scanning electron microscope (HRSEM) (Oberkochen, Germany). Fracture surface samples of the oxide film were prepared by fracturing the film along the edge of the specimens after sectioning a portion of the metal matrix in the area of interest. For the preparation of inner-surface samples, the oxide on one side of the specimens was mechanically ground off, and the metal matrix was subsequently protected with cured epoxy resin. To enhance image quality during SEM observation, a thin layer of gold was sputtered onto the oxide surface. The pristine microstructure of the deposited coatings was analyzed using a glancing-angle X-ray diffractometer equipped with filtered Cu Kα radiation (Y-4Q X-ray diffractometer). The incident X-ray beam angle was set to 2°, with the detected diffraction angle (2θ) ranging from 20° to 90°. The scan rate and step size were set at 2° per minute and 0.01°, respectively.

The adhesion strength of the deposited coatings was evaluated using scratch testing. A WS-2005 scratch tester equipped with a Rockwell C indenter (tip radius R = 0.2 mm, conical angle = 120°) was employed for this purpose (Lanzhou, China). During the test, the normal load applied to the coating gradually increased from 0 N to 200 N at a rate of 50 N/min, with a scratch length of 8 mm. The tester recorded the normal load, acoustic signal, and friction force during the scratch test. Afterward, the surface morphology of the tested specimen was examined using a metallographic microscope. The critical load was determined based on the acoustic signal, friction force data, and the scratched surface image.

## 3. Experimental Results

### 3.1. Surface Morphologies of Coated Zirconium Alloys

**Figure 1:**
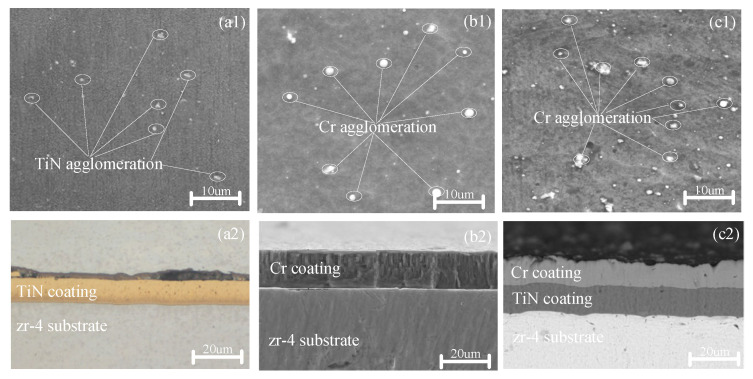
Surface and cross-sectional SEM morphologies of the TiN-, Cr-, and TiN–Cr-coated zirconium alloy samples. (**a1**,**a2**) TiN coating; (**b1**,**b2**) Cr coating; (**c1**,**c2**) TiN- Cr coating.

Figure 1 shows the surface and cross-sectional grain morphologies, along with the corresponding graphical information, of the zirconium alloy samples coated with TiN, Cr, and TiN–Cr prior to oxidation. As depicted in Figure 1, the surfaces of all coated zirconium alloy samples exhibit continuity and density, indicating a well-adhered coating. A notable feature observed across all samples is the presence of surface droplets. These droplets are hypothesized to result from the high current densities used during the deposition process, which lead to the deposition of large molten metal particles onto the substrate. This phenomenon creates a shadowing effect, resulting in droplet formation on the coating surface.

As shown in Figure 1(a2), the TiN coating is uniformly applied with a thickness of approximately 8 μm. The Cr coating, on the other hand, demonstrates excellent adhesion to the Zr substrate, with no observable microcracks or microvoids within the coating or at its interface with the Zr substrate. The microstructure of the Cr coating is characterized by columnar grains with a body-centered cubic (BCC) structure, as depicted in Figure 1(b2). These columnar grains vary in size, with their length oriented perpendicularly to the coating surface, and they tend to elongate as the coating thickness increases.

For the TiN–Cr composite coating, the outer layer consists of Cr with columnar crystal structures, while the inner layer is composed of TiN with equiaxed crystal structures. During the deposition process, ionized Cr^2+^ ions are accelerated, causing secondary bombardment of the target particles. This phenomenon results in a relatively rough surface texture of the coating, as shown in Figure 1(c2).

### 3.2. Film-Substrate Adhesion Evaluation

Table 4 presents the acoustic signal spectrum data for TiN, Cr, and TiN–Cr composite coatings bonded to zirconium alloy substrates. The acoustic signal spectrum, measured by scratch testing, was used to evaluate the film–substrate adhesion of the coated specimens. It was observed that when minor spalling occurs in a coating and the acoustic signal spectrum shows significant fluctuations, the corresponding load reflects the film–substrate adhesion [9]. As shown in Table 3, the average film–substrate adhesion is lowest for TiN coatings, followed by Cr coatings, with TiN–Cr coatings exhibiting the highest adhesion. This is attributed to the fact that TiN coatings, acting as an intermediate layer, possess excellent thermal conductivity. This property mitigates thermal stress between the Cr coatings and the zirconium alloy substrates, thereby improving the film–substrate adhesion in composite coatings.

### 3.3. XRD Analysis

**Figure 2:**
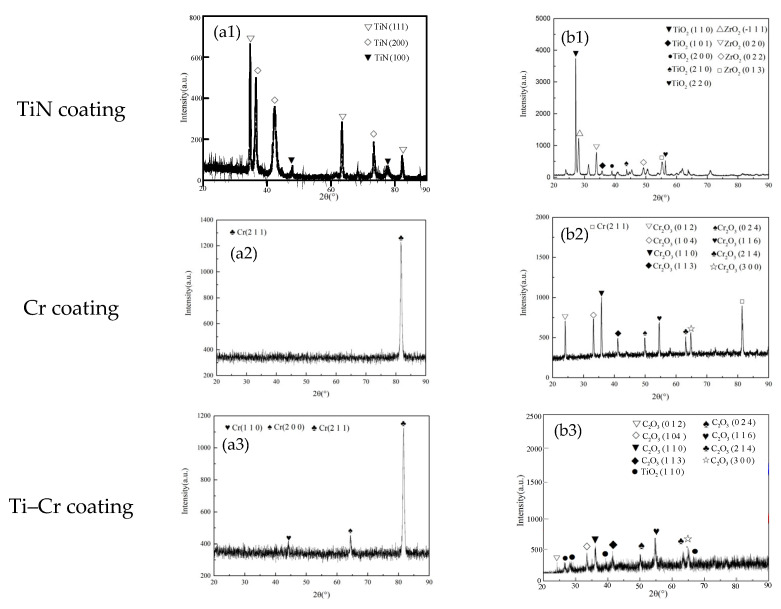
(**a1**–**a3**) XRD spectra of TiN, Cr and Cr–TiN coating before oxidation, respectively; (**b1**–**b3**) XRD spectra of TiN, Cr and Cr–TiN coating after oxidation, respectively.

Figure 2 presents the X-ray diffraction (XRD) patterns of TiN-, Cr-, and Cr–TiN coated zirconium alloys, both before and after 4 h oxidation exposure at 1200 °C. It can be seen that before high-temperature oxidation, the surface of the TiN coating is dominated by the (111) plane, while the surfaces of the Cr and TiN–Cr coatings are mainly dominated by the (211) plane. No oxygen elements were observed on the coating surfaces. As shown in Figure 2(a1–a3), after high-temperature oxidation, the TiN-coated sample exhibited the presence of TiO_2_ and ZrO_2_ phases, with no detectable TiN crystal phase remaining. This observation indicates that the TiN coating on the zirconium alloy underwent complete oxidation, resulting in the formation of a relatively porous and loosely bound TiO_2_ phase, as shown in Figure 2(b1). The term “loose phase” here refers to the oxidation products (TiO_2_ and ZrO_2_) that are not densely packed or strongly adhered to the substrate, leading to a porous and non-compact structure. These loosely bound oxide phases created rapid-oxidation channels, accelerating the oxidation of the underlying zirconium alloy matrix.

In Figure 2(b2), after high-temperature oxidation of the Cr-coated sample, the surface is predominantly composed of the Cr_2_O_3_ phase. Additionally, a significant presence of unoxidized Cr (211) phase was observed, as evidenced by the relatively high intensity of its corresponding diffraction peaks. Notably, no zirconium alloy or its oxide phases were detected on the sample surface in the X-ray diffraction pattern. This result suggests that the high-temperature oxidation of the Cr coating leads to the formation of a dense Cr_2_O_3_ oxide film, which effectively protects the internal zirconium alloy matrix from oxidation.

Following high-temperature oxidation, the surface of the Cr–TiN-coated sample is predominantly composed of Cr_2_O_3_ and TiO_2_ phases, as shown in Figure 2(b3). During the high-temperature oxidation process, chromium (Cr) and titanium (Ti) atoms migrate towards the coating surface, while oxygen (O) atoms diffuse towards the substrate through the grain boundaries of the coating. Initially, the formation of a dense Cr_2_O_3_ layer acts as a barrier, effectively preventing further oxidation and the ingress of O atoms into the coated sample.

However, as high-temperature oxidation progresses, the rate of atomic diffusion increases correspondingly. The Gibbs free energy for the reaction of titanium (Ti) with oxygen (O) to form titanium dioxide (TiO_2_) is −248,628 J/mol, which is significantly more negative than the −167,206 J/mol for the reaction of chromium (Cr) with oxygen to form chromium oxide (Cr_2_O_3_). This thermodynamic preference indicates that oxygen atoms at the surface initially react with the migrated Ti atoms to form stable TiO_2_. Subsequently, the remaining oxygen atoms react with Cr to produce Cr_2_O_3_. This sequence of reactions highlights the significant influence of Ti on the oxidation behavior of the Cr–TiN coating under high-temperature conditions.

### 3.4. Weight Gain

Table 4 presents the thermogravimetric (TG) analysis results for the TiN-, Cr-, and Cr–TiN-coated samples following 4 h oxidation at 1200°C. The weight gain per square decimeter (dm^2^) of the samples, denoted W, was calculated using Equation (1):

W = Δm/S(1)

where Δm represents the difference in sample weight before and after oxidation, and S is the surface area of the sample. As shown in Table 5, the weight gain per unit area for both the uncoated and TiN-coated specimens is relatively high, accompanied by noticeable delamination cracks on the surface, indicating severe oxidation. In contrast, the Cr- and Cr–TiN-coated samples exhibit relatively minor weight gain per unit area, suggesting a more controlled oxidation process. No microcracks are observed on their surfaces. However, small bubbles are present on the surface of the Cr–TiN-coated samples. These bubbles do not appear to compromise the high-temperature performance of the coating.

### 3.5. External Surface Morphologies of Oxide Films

**Figure 3:**
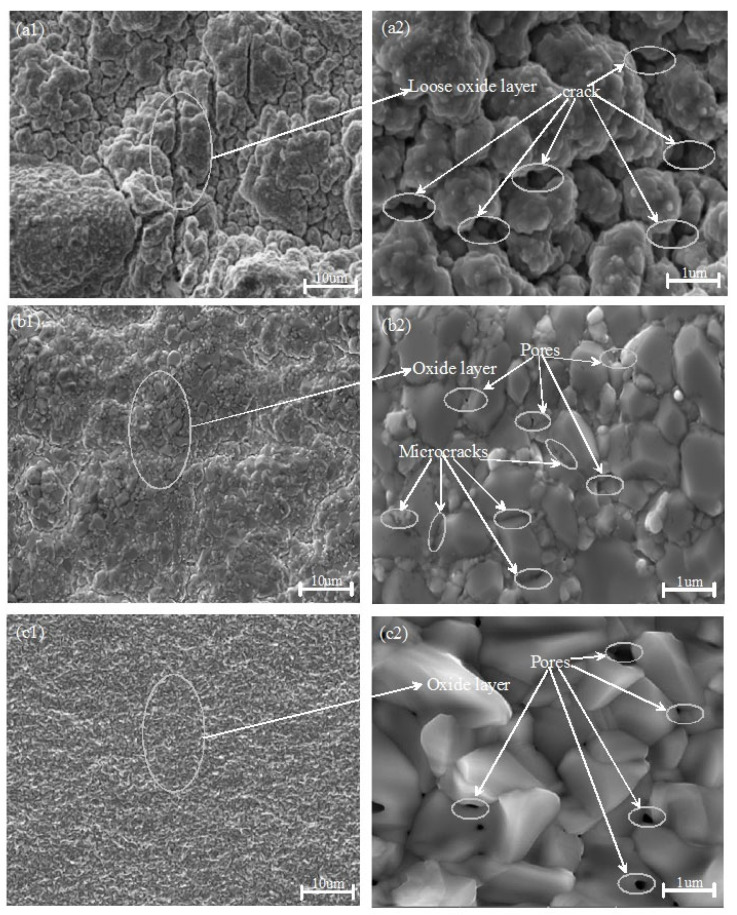
Low (**a1**–**c1**)- and high (**a2**–**c2**)-magnification surface morphologies of the oxide films on the TiN-, Cr-, and Cr–TiN-coated samples after oxidation exposure for 4 h at 1200 °C.

Figure 3 illustrates the typical surface morphologies of zirconium alloy samples coated with TiN, Cr, and Cr–TiN after 4 h oxidation at 1200 °C. Notably, the morphologies of the TiN-, Cr-, and Cr–TiN-coated samples differ significantly. As shown in Figure 3(a1), after high-temperature oxidation, the TiN-coated surface exhibits pronounced wrinkling, along with loose oxide formation and severe cracking. The originally dense TiN coating transforms into fragile particles prone to detachment. In contrast, the surfaces of the Cr- and Cr–TiN-coated samples appear relatively smooth. However, a significant number of voids and microcracks are still observed on the Cr-coated surface. Nevertheless, the formed oxide layer adheres well to the original coating, with no evidence of peeling, as shown in Figure 3(b1,c1).

The observed cracking and detachment of the TiN coating can be attributed to the differing thermal expansion coefficients between the TiN coating and the zirconium alloy matrix. Additionally, at temperatures reaching 1200 °C, the TiN coating rapidly oxidizes, forming a loose TiO_2_ structure. This oxidation process results in surface cracks and the formation of numerous pores on the coating, as shown in Figure 3(a2). In contrast, the Cr- and Cr–TiN-coated zirconium alloys form a dense Cr_2_O_3_ film under high-temperature conditions at 1200 °C, as illustrated in Figure 3(b2,c2). During the cooling process, differences in thermal expansion coefficients among the layers of the sample cause varying degrees of volume shrinkage. This induces internal stress, ultimately resulting in the formation of numerous microcracks and surface undulations. Analysis of the post-high-temperature oxidation surface morphology reveals a significant number of droplets on the surfaces of the Cr- and Cr–TiN-coated samples prepared by multi-arc ion plating. These droplets are initially oxidized in the high-temperature environment, highlighting the complex interplay between the coating materials and the high-temperature oxidation process.

### 3.6. Microstructure Observed from the Cross Section of the Oxide Film

**Figure 4:**
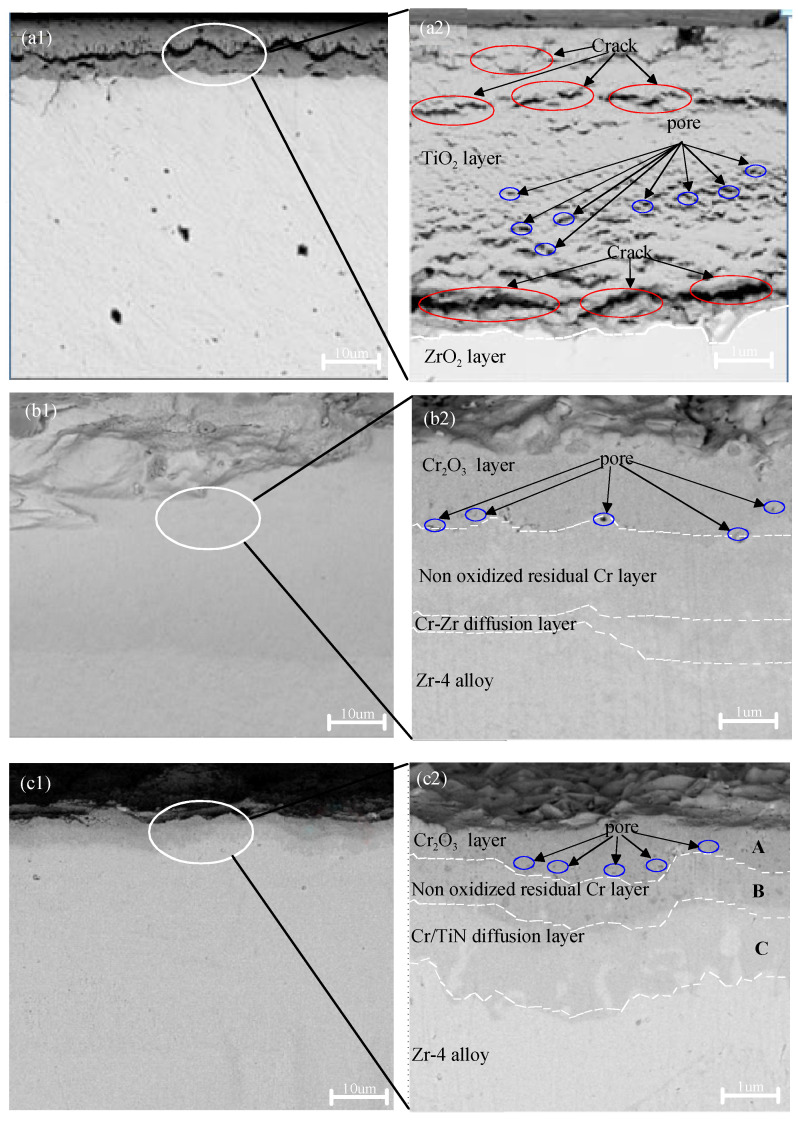
Cross-sectional SEM morphologies of the TiN-, Cr-, and TiN–Cr-coated zirconium alloy samples after oxidation at 1200 °C for 4 h. (**a1**,**a2**) the TiN coating; (**b1**,**b2**) the Cr coating; (**c1**,**c2**) the TiN–Cr coating.

Figure 4 illustrates the cross-sectional morphologies of zirconium alloy samples coated with TiN, Cr, and Cr–TiN, while Table 5 provides the energy spectrum analysis for different regions within the cross sections of these coatings after 4 h oxidation at 1200 °C. As shown in Figure 4(a1,a2), the oxide layer thickness is approximately 40 to 50 μm. A distinct boundary is visible between the oxidized TiN-coating layer and the internal zirconium alloy oxidation layer. The TiN coating appears to be nearly completely oxidized, and the resulting loose oxide layer has detached.

As listed in Table 5, Zr elements were detected on the surface of the TiN coating, accounting for 4.4% of the content. In the middle layer, Zr accounted for approximately 55%. This is attributed to the diffusion of zirconium from the zirconium alloy to the coating surface in a high-temperature environment, forming ZrO_2_. Therefore, it is inferred that both the TiN coating and the adjacent zirconium alloy layer underwent oxidation when the specimen was maintained at 1200 °C for 4 h during the high-temperature oxidation test. During the subsequent air-cooling of the specimen, a significant portion of the oxidized coating is believed to have detached.

Figure 4(b1,b2) shows that the cross section of the specimen can be distinctly divided into four layers: the outermost chromium oxide (Cr_2_O_3_) layer, the intermediate residual Cr layer, the Cr–Zr inter-diffusion layer, and the inner Zr layer. The intermediate residual Cr layer is approximately 4 to 5 μm thick, while the Cr–Zr inter-diffusion layer measures about 2 to 3 μm. Elemental oxygen was detected exclusively in the outermost Cr_2_O_3_ layer, with an oxygen content of 37.4% following high-temperature oxidation. As shown in Table 5, in the residual Cr region, the absence of oxygen elements suggests that the dense chromium oxide layer formed on the surface of the Cr coating acts as a protective barrier for the underlying zirconium alloy substrate. Additionally, a region of mutual diffusion between Cr and Zr elements was identified at the interface between the Cr coating and the zirconium alloy substrate.

In the case of the TiN–Cr bilayer-coated Zr-4 specimen, as shown in Figure 4(c1,c2), gaps and pores were present, allowing for the detection of elemental oxygen at points A, B, and C, as indicated in Table 6. However, due to the significant protective effect of the outermost Cr coating, only a limited amount of oxygen permeated into the interior. As a result, the specimen remained intact, and no detachment was observed on the surface.

**Figure 5:**
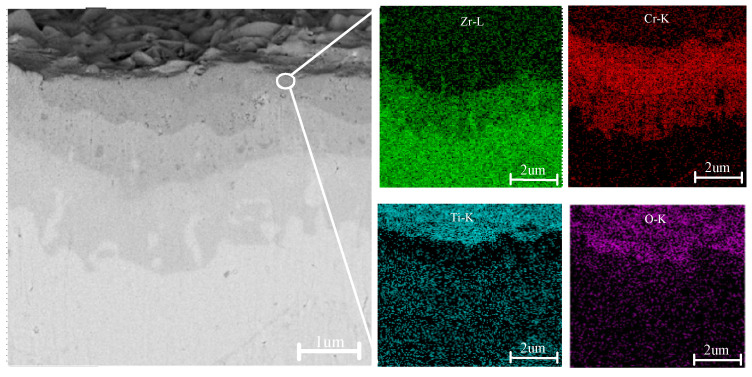
Cross-sectional morphology and EDS surface scan spectrum of the TiN–Cr coating.

Figure 5 presents the cross-sectional morphology and corresponding EDS analysis of a TiN–Cr-coated zirconium alloy sample following high-temperature oxidation. After 4 h of exposure to a high-temperature environment at 1200 °C, the surface coating remains tightly adhered to the Zr-4 alloy matrix, with no signs of microcracks or cavities observed. The cross-sectional view in the upper-right corner of Figure 4(c1) reveals that the sample is stratified into four distinct layers. The darker layers near the upper and lower surfaces of the sample represent oxygen-rich regions, including the oxide layers of Cr and the Zr-4 alloy α-Zr(O). The thickness of the oxygen-rich layer is approximately 1 mm, and within this layer, the sample exhibits numerous cracks and cavities.

## 4. Discussion

**Figure 6:**
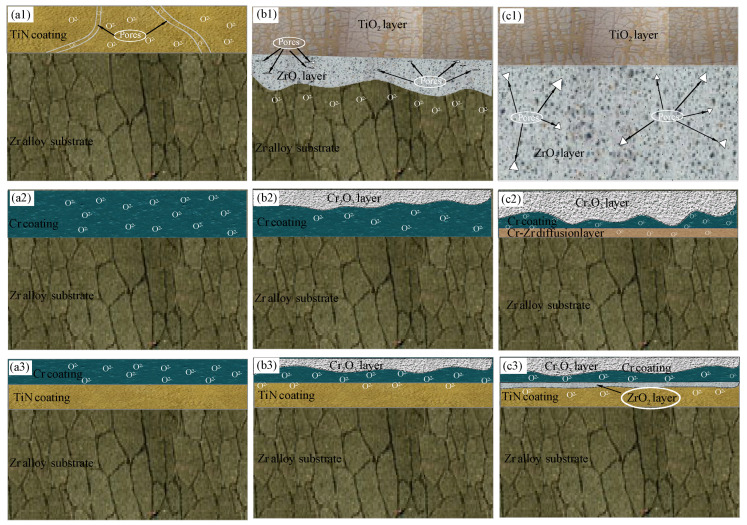
Schematic illustrations of oxidation mechanism of the (**a1**–**c1**) TiN-coated Zr-4 specimen, (**a2**–**c2**) Cr-coated Zr-4 specimen, and (**a3**–**c3**) TiN–Cr-coated Zr-4 specimen.

According to Wagner’s high-temperature oxidation theory, the oxidation behavior of metals at elevated temperatures is primarily governed by the rate of element diffusion. As temperature increases, the diffusion rate escalates, resulting in a higher concentration of defects within the system. This in turn significantly accelerates the oxidation rate. The failure mechanisms of various coatings have been extensively studied in recent scholarly works. In this research, the oxidation processes of TiN, Cr, and TiN–Cr coatings were analyzed by examining the transition from parabolic to linear kinetics and the associated changes in the protective properties of these coatings, as shown in Figure 6.

Figure 6(a1,b1,c1) illustrate the oxidation mechanism of TiN-coated zirconium alloy samples subjected to steam oxidation. After high-temperature oxidation, the TiN-coated zirconium alloy experiences severe oxidation, resulting in a substantial increase in weight. Simultaneously, the oxygen content within the surface oxide layer of the coating significantly increases, while the nitrogen content decreases correspondingly. This phenomenon is attributed to the reaction of TiN with oxygen during the oxidation process, as represented by the following reaction:

2TiN + 2O_2_ = 2TiO_2_ + N_2_

As the oxidation time progresses, the Zr content in the oxide layer increases, indicating that the oxide film of the coating gradually ruptures, allowing oxidation of the underlying substrate to begin. Observations of the oxidized coating samples and corresponding SEM images (see Figure 2) reveal that the samples appear gray-brown at 1200 °C. The morphological images show significant peeling of the film layer, which exposes the substrate and leads to a loss of protective efficacy, as shown in Figure 6(c1).

Figure 6(a2,b2,c2) illustrate the oxidation mechanism of Cr-coated zirconium alloy samples following steam oxidation. The growth mechanism of the Cr_2_O_3_ film during high-temperature oxidation of the Cr coating is characterized by the concurrent outward diffusion of Cr and inward diffusion of oxygen. The oxidation rate is determined by the dynamic equilibrium between these diffusion processes. At 1200 °C, the outward diffusion rate of Cr markedly increases, leading to the formation of a dense protective film on the surface. However, as oxidation continues, excessive internal stress within the film can cause delamination of the outer oxide layer, exposing fresh surfaces for further oxidation [15,16]. Additionally, the distribution of oxygen diffusing into the Cr coating is significantly reduced. This reduction in oxygen diffusion is attributed to the initiation of Zr element growth from the Cr–Zr inter-diffusion layer under high-temperature conditions. Zr elements propagate along the grain boundaries of the columnar crystals within the coating, moving toward the Cr coating. Upon contact with external oxygen through the Cr grain boundaries, the Zr elements, known for their strong oxygen absorption capacity, form channels that facilitate the continuous transport of oxygen to the zirconium alloy matrix. Concurrently, the Cr_2_O_3_ protective film, along with volatile CrO_3_ produced at high temperatures, significantly accelerates the consumption rate of the coating. This process leads to the rapid accumulation and release of thermal stress within the oxide film, resulting in surface cracking, bubbling, and other related phenomena. Although the coating provides a certain level of protection in the short term, these processes ultimately compromise its long-term protective efficacy.

Figure 6(a3,b3,c3) illustrate the oxidation mechanism of TiN–Cr-coated zirconium alloy samples following steam oxidation. The TiN–Cr coating exhibits excellent protective performance in high-temperature steam oxidation, and its protection mechanism is primarily based on the good adhesion between the coating and substrate, the chemical stability of the coating itself, and the dense oxide layer formed on the surface. During the high-temperature oxidation, the growth of the oxide film on the outer layer of the TiN–Cr composite coating is governed by the concurrent outward diffusion of Cr and inward diffusion of oxygen. As oxidation time increases, the consumption rate of the protective Cr_2_O_3_ film and the volatile CrO_3_ coating escalates significantly. This escalation results in the rapid accumulation and release of thermal stress within the oxide film, leading to the formation of oxygen channels. Due to the strong affinity between oxygen (O) atoms and titanium (Ti) atoms, TiO_2_, which possesses very low free energy, is generated [23]. This behavior is attributed to the ability of titanium nitride (TiN) to reduce the oxygen content at the TiN–Cr interface. By minimizing the infiltration of oxygen atoms, TiN serves to protect the zirconium alloy matrix from oxidation, thereby providing a significant protective effect.

Additionally, the combination of TiN and Cr coatings has a synergistic effect. The presence of TiN enhances the adhesion and protective performance of the Cr coating, as shown in Table 3. TiN not only increases the wear resistance of the Cr coating but also forms a transition layer between the TiN and Cr coatings, improving the interface adhesion with the substrate and ensuring that the coating does not easily peel off under high-temperature conditions [13].

In summary, the TiN–Cr coating significantly improves the oxidation resistance of the zirconium alloy substrate in high-temperature steam oxidation environments by forming a dense oxide protective layer, enhancing the adhesion of the coating, and preventing the penetration of steam and oxygen, thereby providing effective protection for the material.

## 5. Conclusions

In this study, zirconium alloy samples coated with TiN, Cr, and TiN–Cr were fabricated using the physical vapor deposition (PVD) technique. The study was conducted in two stages: first analyzing the as-deposited state of the coatings, and then investigating their high-temperature oxidation behavior. The oxidation performance of these coated alloys was systematically examined at 1200 °C for 4 h. The analysis focused on the macroscopic appearance, weight gains, surface features, and cross-sectional morphologies of the samples after high-temperature oxidation. Based on the experimental data, the following principal conclusions were drawn.

The TiN, Cr, and TiN–Cr coatings on the zirconium alloy samples, fabricated using the PVD technique, exhibit continuous and dense surfaces, indicating strong adhesion to the substrate. However, the average film–substrate adhesion strength varies across the different coatings. Specifically, the TiN coating shows the lowest adhesion, the Cr coating demonstrates moderate adhesion, and the TiN–Cr coating achieves the highest adhesion strength.

After high-temperature oxidation, the TiN-coated zirconium alloy undergoes significant surface oxidation, leading to the formation of ZrO_2_ and TiO_2_. Numerous pores and extensive cracking are observed between the coating and the substrate, indicating that the TiN coating is unsuitable as a protective layer for zirconium alloys due to the compromised structural integrity.

Oxidation performance of Cr-coated zirconium alloy: The Cr-coated layer after high-temperature oxidation is stratified into four distinct layers: the outermost Cr_2_O_3_ layer, the intermediate residual Cr layer, the Cr/–Zr interdiffusion layer, and the inner Zr layer. The surface composition of the coating is predominantly Cr_2_O_3_, with no observable precipitation of Zr elements. This composition effectively protects the zirconium alloy from high-temperature oxidation.

Oxidation performance of TiN–Cr-coated zirconium alloy: After high-temperature oxidation, the TiN–Cr-coated layer is also clearly stratified into four layers: the outermost Cr_2_O_3_ layer, the intermediate residual Cr layer, the TiN–Cr interdiffusion layer, and the inner Zr layer. The surface composition of the coating is primarily Cr_2_O_3_, with a minor presence of TiO_2_. While gaps and pores are evident, which facilitate the detection of elemental oxygen, no precipitation of Zr elements is observed. This structure effectively protects the zirconium alloy from high-temperature oxidation.

## Figures and Tables

**Table 1:** Preparation method and characteristics of coated zirconium alloys.

Coating Type	Representative Coating	Preparation Method	Advantages	Disadvantages
Ceramic coating [12,13,14]	MAX phase (TiAlC, Ti_3_SiC_2_)	Cold spraying, magnetron sputtering, etc.	Combined properties of ceramic and metal materials, high bonding strength of film base, good corrosion resistance	Easy to corrode and dissolve in high-temperature water environment, good irradiation performance
	TiAlN, ZrN, TiN	Arc ion plating, pulsed laser deposition, cold spraying, etc.	High hardness, high melting point, high thermal conductivity, good corrosion, general bonding force of film base	Poor high-temperature oxidation resistance, easy to crack, poor irradiation performance
	SiC, ZrC	Ion beam hybrid deposition, etc.	Good irradiation stability, poor film–base adhesion	Poor bonding, easy to peel off the coating at high temperature, irradiation performance stability
Metal or alloy coating [15,16,17,18]	Cr, Al, Mo	3D laser, cathodic arc vapor deposition, cold spraying, magnetron sputtering, etc.	Dense coating, good resistance to high-temperature oxidation, high film–base bonding (~120 N)	Fast mechanical property degradation after irradiation, bad economics for nuclear fuel
	CrAl	Laser cladding, etc.	Good coating density, good resistance to high-temperature oxidation, high film–base bonding force	Coating thickness is large, easy to oxidize and crack under high temperature.
	FeCrAl	Plasma spraying, magnetron sputtering, etc.	Good adhesion, high corrosion resistance	Alloy composition is not easy to control, poor thermal corrosion resistance, easy to dissolve
Composite coating [8,19,20,21,22,23]	Cr-Zr–Cr–Cr-N, Ti_2_AlN–TiAl	Vacuum arc ion plating, high-speed flame spraying, magnetron sputtering, etc.	Dense coating structure, good high temperature oxidation resistance	Insufficient coating density and stability

**Table 2:** Mass percentage content of elements in Zr-4 alloy used in the experiments (mass fraction, %).

Alloy	Sn	Fe	Cr	O	Zr
Zr-4	1.30–1.50	0.18–0.24	0.09–0.12	0.09–0.14	Bal.

**Table 3:** Preparation parameters of the TiN-, Cr-, and TiN–Cr-coated zirconium alloy.

Sputtering Power (W)	Substrate Bias (V)	Substrate Temperature (°C)	Deposition Time (h)			
			TiN	Cr	TiN–Cr	
					TiN	Cr
800	−100	350	10	10	5	5

**Table 4:** Acoustic signal spectra of TiN-, Cr-, and TiN–Cr composite coatings.

Coating Type	Frist	Second	Third	Average
TiN coating	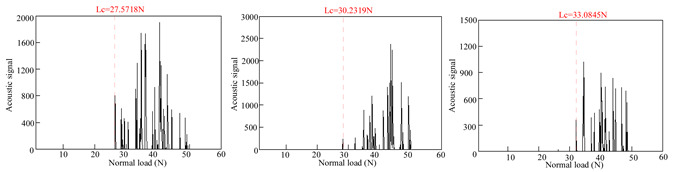			30.29 N
Cr coating	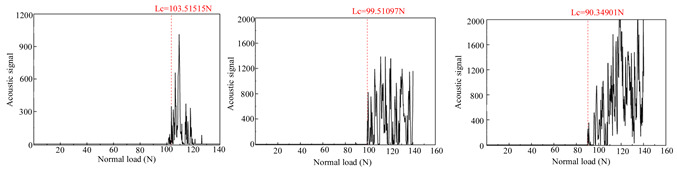			97.79 N
TiN–Cr coating	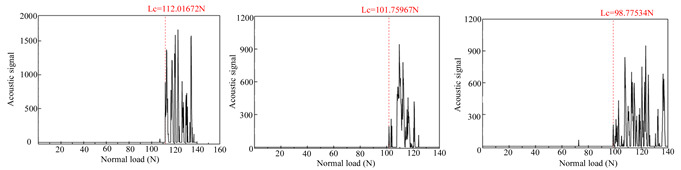			104.18 N

**Table 5:** Weight gain results of high-temperature oxidation of TiN-, Cr-, and Cr–TiN-coated samples.

Material	Weight (g)	Area (dm^2^)	Gain per dm^2^ (g/dm^2^)	Crack	Sample
TiN coating	1.0267	0.0946909	10.84265	Yes	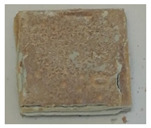
Cr coating	0.6412	0.0942532	6.802952	No	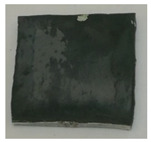
Cr–TiN coating	0.2293	0.0928611	2.46928	No	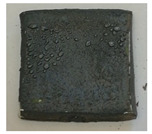
Zr4	1.7512	0.0964472	18.1571	Yes	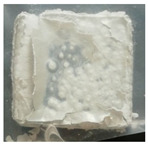

**Table 6:** Energy spectrum analysis of the positions marked in Figure 5.

Coating Type	Mark	Ti	Zr	Cr	O	Others
TiN coating	A	49.9	4.4	--	44.6	1.1
	B	12	50.9	--	36.2	0.9
	C	0.2	74.8	--	25	--
Cr coating	A	--	--	61.4	37.4	1.2
	B	--	--	94.6	3.8	0.6
	C	--	2.3	96.9	--	0.8
Cr–TiN coating	A	14.9	--	35.6	50.6	0.9
	B	18.6	--	73.2	7.9	0.3
	C	61.3	2.6	34.8	0.2	0.5

## Data Availability

The original contributions presented in this study are included in the article. Further inquiries can be directed to the corresponding author.
